# A Multicenter Study on the Prophylactic Application of Antibiotics in Aseptic Operations

**DOI:** 10.5812/ircmj.4914

**Published:** 2013-01-05

**Authors:** Lidao Bao, Tao Shang, Yu Li, Hongping Shen, Zhi Yang, Yi Wang

**Affiliations:** 1 Department of Pharmacy, Inner Mongolia Medical University, Hohhot , P. R. China; 2 Department of Pharmacy, The Third Hospital of Shijiazhuang Affiliated Heibei Medical University, Shijiazhuang, P. R. China; 3 Department of Pharmacy, Yellow River Central Hospital, Zhengzhou, P. R. China; 4 Affiliated T.C.M Hospital of Luzhou Medical College, Luzhou, P. R. China; 5 Department of Neurology, The Second Affiliated Hospital of Jiamusi University, Jiamusi, P. R. China

**Keywords:** Anti-Bacterial Agents, Aseptic, Analysis

*Dear Editor*,

Perioperative prophylactic use of antibiotics aims to prevent bacterial invasion of the wound from entering blood circulation and secondary infection, but antibiotics are mostly not in need unless confronting large operation range, long operation time or operation involving vital organs, foreign body implantation, patients with a high risk of infection factors such as age, diabetes, cancer and others before considering prophylaxis (1, 2). According to the Cruse statistics, the incidence of wound infection was about 1% in aseptic operative procedures (3). However, the perioperative prophylactic use of antibiotic is currently very common in aseptic operative procedures. In the present study, a total of 2483 cases with aseptic operative procedures from October to December in 2011 were randomly selected from five Grade-3 state owned hospitals. The results show the current situation of prophylactic use of antibiotics in six types of aseptic operative procedures and provide evidence for the development of principles for prophylactic antibiotics in aseptic operative procedures and the standardization of prophylactic antibiotics.

2483 completed medical records were incorporated in this study totally (Table 1). The distributed questionnaires, including patient age, gender, disease diagnosis, length of hospital stay, records of disease history and infection risk factors, the preoperative, intraoperative and postoperative use of antibiotics, were retrieved and investigated. The evaluation standards include GAUCP (Guidelines for Antimicrobial Use in Clinical Practice [2004] No. 285, China's Ministry of Health), and No. 38 document (Notification on the clinical application and management of antimicrobial agents issued by the Ministry of Health, 2009) (4). All the 2483 cases of patients (100%) were intravenously infused within 20-30 min during operation, which is reasonable because slow dropping of the massive diluted infusion is not suitable for achieving the effective drug concentration (5).

**Table 1:** General Information of the Cases

	Thyroid surgery	Breast	Vascular surgical	Abdominal external hernia	Splenectomy	Closed fractures	Sum total
**Gender**							
Male	258		133	88	51	507	1037
Female	419	504	137	66	47	273	1446
**Age range (Average)**	14-67 (46.29)	26-74 (64.87)	19-72 (57.19)	8-75 (38.26)	48-70 (54.98)	12-82 (54.10)	
**Length of stay, day**	16.2	7.9	7.1	6.8	14.7	22.6	
**Infection risk factors**							
> 70 years	2	2		2		5	11
Malignancy		3			1		4
Diabetes mellitus	1	8	2	2	1	11	25
**Fervescence in perioperative period**		5		1	3		9

1616 (65.08%) out of the 2483 cases were treated with antibiotics in the perioperative period, including cephalosporins, fluoroquinolones, nitroimidazoles and penicillin. Major patients did not show indications concerning the use of antibiotics, and the ranges of some operations (benign breast tumor resection, partial thyroidectomy, hernia repair) were narrow in which antibiotics were also used for the operations within 1 h. Obviously, prophylactic medication indications were not strictly mastered, and the drug use range was expanded, which will not only enable the bacteria to be drug resistant easily, but also increase the economic burden of the patients.

Combined antibiotics are commonly not recommended for general aseptic operations (6), and the combination of three types of antibiotics is only allowed under special circumstances to prevent infections. In the investigated 837 cases using antibiotics, single antibiotics were used in 658 cases, (26.50%), two drugs were utilized in 172 cases (6.93%), and three drugs were combined in 7 cases (0.28%). Antimicrobial agents were changed for 41 patients (1.65%) during operation. The combination of more types of drugs may lead to higher incidence rates of side effects (7).

Moreover, guiding principles emphasize that drugs should be given in 0.5-2 h before operation or at the beginning of anesthesia for aseptic operation patients. If the operation time is longer than 3 h or the blood loss quantity is higher than 1500 mL, a second portion of drugs should be given. Effective coverage time of antimicrobials should include the entire operation and 4 h after operation. The overall prophylactic medication time should be shorter than 24 h, which may be extended for 48 h under special circumstances. For the aseptic operation with relatively short operation time (< 2 h), treating the patients with preoperative medication for once is sufficient.4 In our study, 28 patients were treated for more than 2 h before operation (1.13%), 80 patients were treated for 0.5-2 h of administration, accounting for 3.22%. After delivery in 729 cases, accounting for 29.36%; after 24 hours of drug discontinuation in 515 cases, accounting for 20.74%; stopped medicine in 24-48 hours in 149 cases, accounting for 6%; more than 48 hours in 65 cases, accounting for 2.62%. It has been previously reported that even if antibiotics were applied for several times or several days after operation, the infection rates could still not be reduced. Meanwhile, using antibiotics continuously for the uninfected incisions within 48 h would lead to drug resistance that may induce uncontrollable infections (8). Thus, the administration time of antibiotics in the study is too long.

The irrational phenomena found in the multicenter study herein have attracted particular attention of the hospital regulatory departments. Therefore, series of measures have been released to manage and reinforce the rational use of antibiotics, establish and perfect the management system of the use of antibiotics. Furthermore, we have developed a warning system for the clinical use of antibiotics, a classified management system for antibiotics, a management regulation for the prevention and use of antibiotics during surgical operations, and a management regulation for the prevention and use of antibiotics during aseptic operations. 
